# The anti-tumor effects of cetuximab in combination with VTX-2337 are T cell dependent

**DOI:** 10.1038/s41598-020-80957-z

**Published:** 2021-01-15

**Authors:** Yinwen Cheng, Nicholas Borcherding, Ayomide Ogunsakin, Caitlin D. Lemke-Miltner, Katherine N. Gibson-Corley, Anand Rajan, Allen B. Choi, Wattawan Wongpattaraworakul, Carlos H. F. Chan, Aliasger K. Salem, George J. Weiner, Andrean L. Simons

**Affiliations:** 1 Interdisciplinary Graduate Program in Human Toxicology, University of Iowa, Iowa City, IA USA; 2 Department of Pathology, University of Iowa, Iowa City, IA USA; 3 Holden Comprehensive Cancer Center, University of Iowa, Iowa City, IA USA; 4 Iowa Medical Scientist Training Program, Carver College of Medicine, University of Iowa, Iowa City, IA USA; 5 Department of Biochemistry, Lincoln University, Lincoln University, PA USA; 6 Department of Internal Medicine, University of Iowa, Iowa City, IA USA; 7 Department of Oral Pathology, Radiology and Medicine, College of Dentistry, University of Iowa, Iowa City, IA USA; 8 Department of Surgery, University of Iowa, Iowa City, IA USA; 9 Division of Pharmaceutics and Translational Therapeutics, College of Pharmacy, University of Iowa, Iowa City, IA USA

**Keywords:** Oncology, Cancer therapy, Head and neck cancer, Oral cancer, Tumour immunology, Head and neck cancer, Oral cancer

## Abstract

The Toll-like receptor 8 (TLR8) agonist VTX-2337 (motolimod) is an anti-cancer immunotherapeutic agent that is believed to augment natural killer (NK) and dendritic cell (DC) activity. The goal of this work is to examine the role of TLR8 expression/activity in head and neck squamous cell carcinoma (HNSCC) to facilitate the prediction of responders to VTX-2337-based therapy. The prognostic role of TLR8 expression in HNSCC patients was assessed by TCGA and tissue microarray analyses. The anti-tumor effect of VTX-2337 was determined in SCCVII/C3H, mEERL/C57Bl/6 and TUBO-human EGFR/BALB/c syngeneic mouse models. The effect of combined VTX-2337 and cetuximab treatment on tumor growth, survival and immune cell recruitment was assessed. TLR8 expression was associated with CD8+ T cell infiltration and favorable survival outcomes. VTX-2337 delayed tumor growth in all 3 syngeneic mouse models and significantly increased the survival of cetuximab-treated mice. The anti-tumor effects of VTX-2337+ cetuximab were accompanied by increased splenic lymphoid DCs and IFNγ+ CD4+ and tumor-specific CD8+ T cells. Depletion of CD4+ T cells, CD8+ T cells and NK cells were all able to abolish the anti-tumor effect of VTX-2337+ cetuximab. Altogether, VTX-2337 remains promising as an adjuvant for cetuximab-based therapy however patients with high TLR8 expression may be more likely to derive benefit from this drug combination compared to patients with low TLR8 expression.

## Introduction

Activation of immune responses through toll-like receptor (TLR) stimulation is currently being investigated as an alternate and adjuvant to standard cancer therapies^1^. TLRs are transmembrane proteins that are expressed on the cell’s surface and in endosomes^2^. TLRs activate the innate immune system by binding with PAMPs/DAMPs and largely determine the development of the adaptive immune response^2,3^. Ten TLRs (TLR1-10) have been identified in humans. TLR7-9 in particular recognize virus-associated nucleic acids, are localized in endosomes^2,4^, and signals through the myeloid differentiation factor 88 (MyD88)-dependent pathway which ultimately leads to NF-κB activation^4–6^.

The role of TLR stimulation in cancer therapy is context specific in that both pro-tumorigenic and anti-tumor responses are observed^7,8^. However, agonists for TLR 7–9 have clearly demonstrated anti-tumor responses in various preclinical models and have sparked interest in the cancer immunotherapy field^9,10^. With respect to HNSCC therapy, TLR8 agonists have shown promise^11–14^. TLR8 is mostly expressed in monocytes, macrophages and dendritic cells (DCs). The TLR8 agonist VTX-2337 (motolimod) activates monocytes and DCs to produce IL-12 and TNFα, which promote Th1 immune responses^15^. VTX-2337 also activates natural killer (NK) cells^12–14^ which strongly justified the investigation of VTX-2337 in combination with the epidermal growth factor receptor (EGFR) inhibitor cetuximab, which is well known to induce NK cell-mediated antibody-dependent cell-mediated cytotoxicity (ADCC)^16^. Promising results were observed with VTX-2337 in combination with cetuximab in a phase 1b clinical trial for previously untreated HNSCC^11^. Unfortunately, in a phase 2 clinical trial (Active8, NCT01836029), VTX-2337 failed to improve overall or progression free survival when combined with the EXTREME regimen (cetuximab + cisplatin/5-fluorouracil) in R/M HNSCC patients^17^. The goal of this study is to further investigate this drug combination (cetuximab + VTX-2337) to determine possible predictors of response.

## Methods

### TCGA analysis

A dataset of gene expression of 522 HNSCC patients (TCGA_HNSC_exp_HiSeqV2-2015-02-24) along with the corresponding clinical outcomes were downloaded from the Cancer Genome Atlas (TCGA), using Xena Functional Genomics Explorer (University of California—Santa Cruz). Patients were divided into tertiles according to their *TLR8* gene expression levels and labelled as “high” (n = 91), “medium” (n = 316) and “low” (n = 115) *TLR8* gene expression. Separation of *TLR8* expression levels into these 3 groups were determined based on gene-level transcription estimates reported in the database in log2(x + 1) transformed RSEM (RNA-Seq by Expectation–Maximization) normalized count. Level 0–4 was defined as “low” expression of TLR8, 4–7 was defined as “medium” expression and 7–10 was defined as “high” expression. The 3 expression groups were analyzed for differences in overall survival (using Kaplan Meier curves) and levels of activated CD4+ T cells and CD8+ T cells. Expression of immune cell population in tumors were estimated using CIBERSORT algorithm based on gene expression for 22 types of flow-purified immune cell population^18^.

### HNSCC TMA analysis

HNSCC tissue microarrays (TMAs) were constructed from formalin-fixed paraffin-embedded oral squamous cell carcinomas (OSCCs) from 146 OSCC patients as previously described in Rajan et al.^19^. Tumor samples were obtained from the archives of the Department of Pathology at the University of Iowa Hospitals and Clinics. All experiments were performed after approval from the University of Iowa Institutional Review Board and all experiments were performed in accordance with guidelines set out by the University of Iowa. Informed consent was obtained from each subject. Only 5 of these tumors were human papilloma virus (HPV)-positive. Sections of tumor (4 µm) were obtained from the TMAs on poly-l-lysine-coated glass slides and subjected to antigen retrieval using pH 6 citrate buffer at 110˚C for 15 min (for TLR8 staining) or pH 9 Tris/EDTA buffer at 95 °C for 15 min (for CD8 staining). Sections were washed in Dako Buffer (Agilent; Santa Clara, CA), endogenous peroxidase quenched by incubation with 3% hydrogen peroxide in 100% methanol for 8 min, sections washed in buffer, incubated with Dako Background Buster for 60 min, then incubated for 60 min at room temperature in rabbit polyclonal anti-TLR8 antibody (Atlas #HPA0016008) diluted 1:1000 or anti-CD8 antibody (Dako #M7103) diluted 1:100 in Dako buffer. After further washes, sections were processed with the Dako rabbit Envision system for 30 min, washed and counterstained with hematoxylin. Slides were examined by pathologists (KNG) and semi-quantitatively scored based on the following rubric: 0 = no immunoreactivity (IR), 1 = rare to scattered IR cells, 2 = multifocal IR cells, 3 = coalescing/clumping foci of IR cells, 4 = bands and sheets of IR cells.

### Cell lines and reagents

The SQ20B cell line was a gift from Dr. Anjali Gupta (The University of Iowa). The SCCVII cell line was a gift from Dr. George Weiner. The TUBO-human EGFR (TUBO-hEGFR) cell line was gifted to our lab from Dr. Yang-Xin Fu (Department of Pathology, University of Chicago, IL)^20^, and the mEERL cell line was a gift from Dr. Paola Vermeer (Department of Surgery, University of South Dakota Sanford School of Medicine, SD)^21^. All cell lines were authenticated by short tandem repeat profiling and used over a course of no more than 3 months after resuscitation of frozen aliquots. All cell lines were cultured in Dulbecco’s Modified Eagle’s Medium (DMEM) containing 10% fetal bovine serum (FBS) and 0.1% gentamicin, except for mEERL which was cultured in DMEM supplemented with 40.5% 1:1 DMEM/Hams F12, 10% FBS, 0.1% gentamicin, 0.005% hydrocortisone, 0.05% transferrin, 0.05% insulin, 0.0014% tri-iodo-thyronine and 0.005% EGF. Cells were cultured in a humidified incubator at 37 °C and 5% CO_2_. Motolimod (VTX-2337) was purchased from AdooQ Bioscience (Irvine, CA). Cetuximab was obtained from the inpatient pharmacy at the University of Iowa Hospitals and Clinics. Human immunoglobulin G (IgG) was purchased from Sigma-Aldrich (St. Louis, MO).

### In vitro immune cell activation

PBMCs were isolated from healthy donor blood obtained from the DeGowin Blood Center (University of Iowa Hospitals and Clinics) by Ficoll density gradient centrifugation. PBMCs were treated with VTX-2337 (0.1–2 μM) for 24 h before analysis of cytokines (IFNγ, TNFα and IL-1β) in cell culture media using Human Quantikine ELISA kits (R&D systems). For the co-culture systems, SQ20B HNSCC cells were 1:1 cultured with human PBMCs and treated with VTX-2337 (2 µM) and/or cetuximab (50 μg/mL) for 24 h. Control cells were treated with human IgG_1_ (50 μg/mL). Culture media containing PBMCs were stained with anti-CD45, anti-CD3, anti-CD16, anti-CD54, anti-CD4, anti-CD8, CD19, CD56, and anti-CD69 antibodies conjugated to different fluorochromes. PBMCs were then analyzed for levels of activated CD4+ T cells (CD45+ CD3+ CD19−CD4+ CD69+), activated CD8+ T cells (CD45+ CD3+ CD19−CD8a+ CD69+) and NK cells (CD45+ CD3−CD16−CD56+ CD54+ CD16−) by flow cytometry.

### In vivo studies

Female C3H/HeJ and C57BL/6J mice (6–8 weeks) were purchased from Jackson Laboratory (Bar Harbor, ME). Female, male, ovariectomized and SHAM female BALB/c mice (7–10 weeks) were purchased from Envigo Laboratories (Huntingdon, Cambridgeshire, United Kingdom). All procedures were approved by the IACUC committee of the University of Iowa and conformed to the guidelines established by the National Institutes of Health (NIH). SCCVII cells (1 × 10^6^ cells/mouse), mEERL cells (1 × 10^6^ cells/mouse) and TUBO-hEGFR cells (5 × 10^5^ cells/mouse) were inoculated into the right flank of C3H/HeJ, C57BL/6 mice and BALB/c mice respectively by subcutaneous injection of 0.1 mL aliquots of saline containing cancer cells into the right flank. Treatment was initiated when the diameter of tumors reached 3–5 mm in any dimension. C3H mice bearing SCCVII tumors, C57BL/6 mice bearing mEERL tumors, and BALB/c mice bearing TUBO-hEGFR tumors were randomized into control (PBS, i.p. every other day, n = 5–9 mice/group) and VTX-2337 (VTX, 1 mg/kg i.p. every other day, n = 5–9 mice/group). For the combined drug treatment experiments, male and female TUBO-hEGFR-bearing BALB/c mice (n = 10 mice/group, 5 male/5 female) were randomized into the following treatment groups: VTX-2337 (VTX) group—1 mg/kg i.p. every other day, Cetuximab (CTX) group—8 mg/kg i.p. twice per week, and VTX-2337 + Cetuximab (VTX + CTX) group—VTX-2337 and CTX at the doses and treatment schedules described above. Control (IgG) mice were administered human IgG at 8 mg/kg i.p. twice per week and PBS i.p. every other day. The duration of treatment was a maximum of 2 weeks. Female BALB/c mice (n = 9–10 mice/group) bearing TUBO-EGFR tumors were administered VTX + CTX (as described above) with or without murine anti-CD4 (100 μg, clone GK1.5, Bioxcell), anti-CD8 (300 μg, clone 53–6.7, Bioxcell) and anti–asialo GM1 (50 μg, Thermo Fisher Scientific). The immune cell depleting antibodies were given 3 days and 1 day before tumor inoculation and twice per week after tumor inoculation.

### Analysis of circulating cytokines and immune cell recruitment

Blood, tumors and spleens were harvested immediately after euthanization from a subset (n = 4–5) of mice. The concentrations of proinflammatory analytes cytokines in the mouse sera were determined using a mouse Bio-Plex 23 panel assay, as per the manufacturer's instructions (Bio-Rad Hercules, California, USA). Single cell suspensions were obtained from digested tumors and spleens and cells were stained with cocktails of antibodies. T cells were defined as CD45+ CD19−CD3+ CD4/CD8+ lymphocytes; NK cells were defined as CD45+ CD3−CD19−CD122+ DX5+ lymphocytes and activated NK cells were defined as CD69+ KLRG1+ . Dendritic cells were defined as CD45+ CD3−CD19−NKp46−CD11c+ immune cells. Cells were analyzed by flow cytometry.

### Statistics

Statistical analysis was carried out using GraphPad Prism version 8 for Windows (GraphPad Software, San Diego, CA). Kaplan–Meier survival curves were generated to illustrate the different survival rates over time. Differences in survival were determined by Log-rank (Mantel–Cox) test. Fisher's exact test and Chi-square test was used to analyze any associations of *TLR8* expression with patient characteristics. Pearson’s correlation test was used to analyze associations between *TLR8* and immune genes. The associations between TLR8 protein expression with clinicopathological features were tested using the generalized linear modeling (GLM) framework and the Kruskal–Wallis test. One-way ANOVA with Tukey post-tests was used to compare the difference between at least three groups. Linear regression models were used to estimate the group-specific change in tumor growth curves. Statistical significance was defined as p < 0.05.

### Ethics approval and consent to participate

All data obtained from the TCGA are completely anonymized and available to the public, therefore, further approval for its use was not required. Analysis of TMAs and associated clinical data was approved by the University of Iowa Institutional Review Board.

## Results

### Increased TLR8 gene expression is associated with favorable overall survival

**Figure 1:**
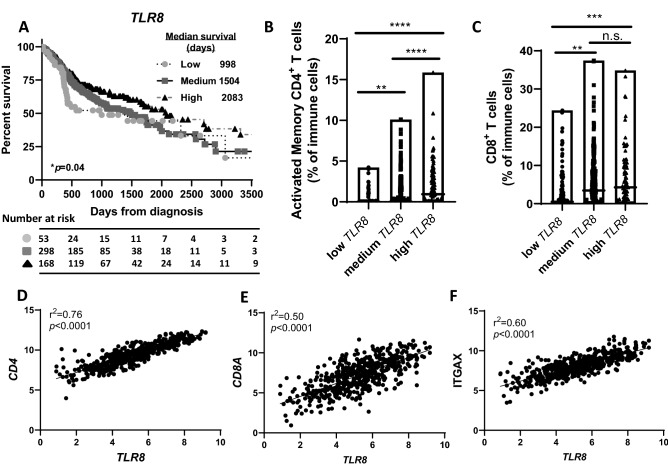
TLR8 gene expression is associated with HNSCC patient survival. (**A**) Shown are Kaplan–Meier survival curves comparing overall survival of HNSCC patients (n = 522) from the TCGA database according to high (n = 91), medium (n = 316) or low (n = 115) *TLR8* expression. (**B**, **C**): Dot-box plots illustrate the percentage of activated memory CD4^+^ (**B**) and CD8^+^ (**C**) tumor infiltrating T cells in HNSCC patients with high, medium and low *TLR8* expression from (**A**). Error bars represent standard deviation of the mean. (**D**–**F**) Correlation analysis (Pearson’s correlation tests) between TLR8 and *CD4* (**D**), *CD8A* (**E**) and ITGAX (**F**) of all HNSCC patients from (**A**). **p < 0.01, ***p < 0.001, ****p < 0.0001, n.s.-non-significant (One-way ANOVA, Tukey’s multiple comparison test).

HNSCC patients (n = 522) from the TCGA database were divided into tertiles according to their *TLR8* gene expression levels and labelled as “high” (n = 91), “medium” (n = 316) and “low” (n = 115) *TLR8* gene expression. The resultant survival curves demonstrated that high *TLR8* expression was associated with a more favorable survival outcome (median survival = 2083 days) compared to medium (median survival = 1504 days) and low (median survival = 998 days) *TLR8* expression (Fig. 1A). Analysis of corresponding patient characteristics and available clinicopathological data indicated no differences between the 3 patient cohorts on the basis of age, clinical stage, or primary/followup therapy outcomes (Supplemental Table 1). Significant gender differences were observed due to the majority of the HNSCC patients being male (74% male vs 26% female), however a significantly higher percentage of females were observed in the high *TLR8* expression patient cohort (39.6%) compared to low *TLR8* expression (18%), while the percentage of males was significantly higher in the low *TLR8* expression group (81.7%) compared to high *TLR8* (60.4%) (Supplemental Table 1). Differences were observed on the HPV status however this was due to the high number of tumors with an “unknown” HPV status in each patient cohort. An analysis of select immune genes revealed increased levels of *CD4* (Fig. 1B) and *CD8* (Fig. 1C) in the high *TLR8* expression group compared to moderate and low *TLR8* expression. Pearson’s correlation analysis showed significant correlations between *TLR8* expression and *CD4* (Fig. 1D), *CD8A* (Fig. 1E) and *ITGAX* expression (Fig. 1F) in the HNSCCs analyzed. Altogether, high *TLR8* is associated with favorable survival outcomes compared to moderate/low TLR8 expression, which may be due to the increased presence of CD4+ and CD8+ T cells.

### Increased TLR8 protein expression is associated with favorable overall survival

**Figure 2:**
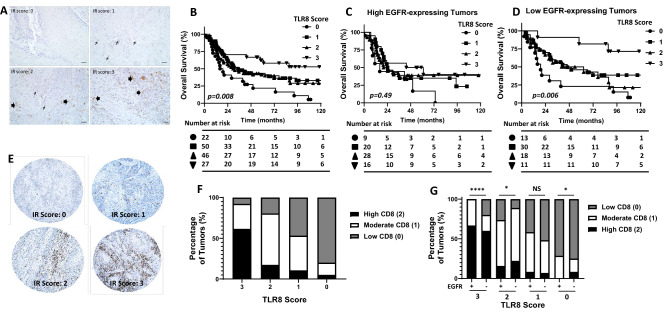
TLR8 protein expression is associated with HNSCC patient survival. (**A**) Representative examples of TLR8 immunostaining and expression scores in HNSCCs. (**B**) Kaplan–Meier estimates of overall survival according to TLR8 expression score(s). (**C**, **D**) Kaplan–Meier estimates of overall survival according to TLR8 expression score(s) in low EGFR-expressing (**C**) and high EGFR-expressing (**D**) HNSCCs. (**E**) Representative examples of CD8 immunostaining and expression scores in HNSCCs from (**A**). F: Percentage of tumors with high, moderate or low CD8 immunostaining based on TLR8 expression scores. G: Percentage of tumors with high, moderate or low CD8 immunostaining based on TLR8 + EGFR expression. IR: immunoreactivity. *p < 0.05, ****p < 0.0001, n.s.-non-significant (Fisher’s exact test).

To determine if the association between *TLR8* gene expression and survival outcomes in Fig. 1 is similar for TLR8 protein expression, we utilized TMAs constructed from 146 tumors from the oral cavity ^19^. Examples of IHC images of TLR8 staining and scores are shown in Fig. 2A. Patients were separated into 4 cohorts according to TLR8 staining intensity (0,1,2 and 3, Fig. 2A). There were no significant associations observed for TLR8 expression with patient gender, age, tumor (T) stage, degree of differentiation, perineural invasion, lymphovascular invasion, bone invasion or post-surgery radiotherapy (Table 1). Associations were observed between TLR8 expression and smoking history, tumor site and nodal (n) status (Table 1). Tumors with high TLR8 expression (TLR8 score = 3) were significantly associated with a more favorable survival outcome compared to lower TLR8-expressing tumors (Fig. 2B). There was no association between TLR8 expression and progression-free survival (data not shown). Given that EGFR is a well-known important prognostic marker in HNSCC^22^, we separated the tumors into high EGFR-expressing tumors (n = 73) and low/no EGFR-expressing tumors (n = 72) as previously described^19^. We observed no differences in overall survival based on TLR8 score in high EGFR-expressing tumors (p = 0.49, Fig. 2C) however in low/no EGFR-expressing tumors, high TLR8 expression was significantly associated with a more favorable survival (p = 0.006) compared to lower TLR8 expression (Fig. 2D). We also probed if higher CD8 expression would be observed in tumors with higher TLR8 expression scores. Examples of CD8 staining and scores are shown in Fig. 2E. Patients were initially separated into 4 cohorts according to CD8 staining intensity (0,1,2 and 3, Fig. 2E), however there was only 1 tumor out of the 145 tumors that was assigned a CD8 score of 3 (Fig. 2E bottom right) and was thus included in the cohort assigned a CD8 score of 2. Indeed, there was a clear and significant difference (p < 0.0001) in CD8 expression according to TLR8 score where tumors with a TLR8 expression score of 3 had the highest percentage of tumors with high CD8 (score of 2) expression (p < 0.001, Fig. 2F). Additionally, tumors with no TLR8 expression had the highest percentage of tumors with no CD8 (score of 0) expression (p < 0.001, Fig. 2F). Furthermore, EGFR status did not influence the percentage of tumors with high CD8 expression in the high TLR8 (score of 3) cohort (Fig. 2G). Altogether these results suggest that high TLR8 expression is associated with a favorable survival outcome in HNSCC patients, but this finding may only be relevant in low or no EGFR-expressing tumors. These results also support the beneficial role of CD8+ T cells on overall survival in high TLR8 expressing HNSCC tumors.

**Table 1:** Patient characteristics by TLR8 staining.

TLR8 Score						
Characteristics	Total (n)	3	2	1	0	*p-value*
**Number of evaluable subjects: n**	145	27	46	50	22	
**Sex: n (%)**						
Male	84	14 (17)	29 (35)	27 (32)	14 (17)	***p = 0.52***
Female	61	13 (21)	17 (28)	23 (38)	8 (13)	
**Age: average (range)**	62 (19–93)	63 (20–93)	58 (19–89)	64 (37–93)	61 (35–80)	***p = 0.27***
Male	58 (31–81)	57 (31–70)	57 (40–81)	60 (37–76)	60 (37–77)	***p = 0.93***
Female	66 (19–33)	70 (20–93)	61 (19–89)	69 (37–93)	64 (35–80)	
**Smoking history: n (%)**						
Active smoker	57	3 (5)	20 (35)	24 (42)	10 (18)	
Never smoker	51	11 (22)	15 (29)	18 (35)	7 (14)	
Quit < 10 years	13	6 (46)	3 (23)	2 (15)	2 (15)	***p < 0.001***
Quit > 10 years	18	5 (28)	5 (28)	5 (28)	3 (17)	
Tobacco chewer	6	2 (33)	3 (50)	1 (17)	0 (0)	
**Tumor site: n (%)**						
Alveolus	23	5 (22)	6 (26)	10 (44)	2 (9)	
Floor of the mouth	32	2 (6)	12 (38)	14 (44)	4 (12.5)	***p = 0.004***
Oral tongue	55	13 (24)	17 (31)	16 (29)	9 (16)	
Other	35	7 (20)	11 (31)	10 (29)	7 (20)	
**T Stage: n (%)**						
T1	43	7 (16)	14 (33)	14 (33)	8 (19)	
T2	43	10 (23)	13 (30)	13 (30)	7 (16)	***p = 0.61***
T3/T4	59	10 (17)	19 (32)	23 (39)	7 (12)	
**N Stage: n (%)**						
N0	74	17 (23)	23 (31)	24 (32)	10 (14)	
N1/2a	29	5 (17)	7 (24)	8 (28)	9 (31)	***p < 0.001***
N2b/2c/3	42	5 (12)	16 (38)	18 (43)	3 (7)	
**Differentiation: n (%)**						
Well	17	2 (11)	6 (36)	7 (41)	2 (12)	
Moderate	91	16 (18)	29 (32)	33 (36)	13 (14)	***p = 0.17***
Poor	37	9 (24)	11 (30)	10 (27)	7 (19)	
**Perineural invasion: n (%)**						
Yes	73	11 (15)	23 (32)	25 (34)	14 (19)	***p = 0.32***
No	72	16 (22)	23 (32)	25 (35)	8 (11)	
**Lymphovascular invasion: n (%)**						
Yes	54	6 (11)	20 (37)	20 (37)	8 (15)	***p = 0.14***
No	91	21 (23)	26 (29)	30 (33)	14 (17)	
**Bone invasion: n (%)**						
Yes	44	6 (14)	17 (39)	16 (37)	5 (11)	***p = 0.23***
No	101	21 (21)	29 (29)	34 (34)	17 (17)	
**Radiotherapy: n (%)**						
Yes	81	13 (16)	25 (31)	29 (36)	14 (17)	***p = 0.59***
No	64	14 (21)	21 (33)	20 (31)	8 (13)	
**Chemotherapy: n (%)**						
Yes	23	1 (4)	6 (26)	9 (39)	7 (30)	***p = 0.0002***
No	122	26 (21)	40 (33)	41 (34)	15 (12)	

### VTX-2337 induces immune cell activation

**Figure 3:**
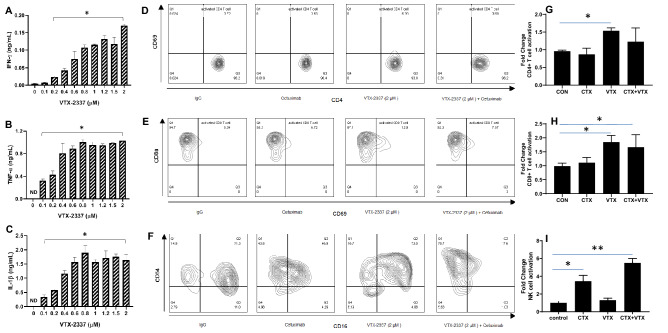
A TLR8 agonist stimulates immune cell activity. (**A**–**C**) Peripheral blood mononuclear cells (PBMCs) were treated with serial dilutions of VTX-2337 for 24 h, then cell culture media harvested for analysis of IFN-γ (**A**), TNFα (**B**) and IL-1β (**C**) by ELISA. *p < 0.01 versus 0 µM. (**D**–**I**) SQ20B cells were co-cultured with PBMCs, treated with VTX and/or cetuximab (CTX) for 24 h, then activated CD4^+^ (**D**, **G**), CD8^+^ (**E**, **H**), and NK cells (**F**, **I**) analyzed by flow cytometry. Human IgG_1_ was used as a control (CON). Bar graphs shown in (**G**–**I**) represent the mean of n = 3 experiments. Error bars represent standard deviation from the mean. *p < 0.05 versus CON, **p < 0.01 versus CON (One-way ANOVA, Tukey’s multiple comparison test).

VTX-2337 (VTX) is believed to promote Th1 immune responses. Indeed the incubation of human PBMCs with serial dilutions of VTX-2337 triggered the secretion of IFNγ (Fig. 3A), TNFα (Fig. 3B) and IL-1β secretion from PBMCs (Fig. 3C). EGFR-expressing SQ20B HNSCC cells were then co-cultured with human PBMCs and treated with VTX alone and in combination with CTX. We found a modest but significant increase in activated CD4 + and CD8 + T cells with VTX-2337 but no enhanced effect with CTX treatment was observed (Fig. 3D,E,G,H). VTX did not increase the number of activated NK cells, but when combined with CTX, significantly increased activated NK cells about fivefold compared with controls (Fig. 3F,I). These results suggest that VTX can increase the activation of T cells and stimulate the release of mediators involved in immune response.

### VTX-2337 suppresses tumor growth in murine syngeneic models

**Figure 4:**
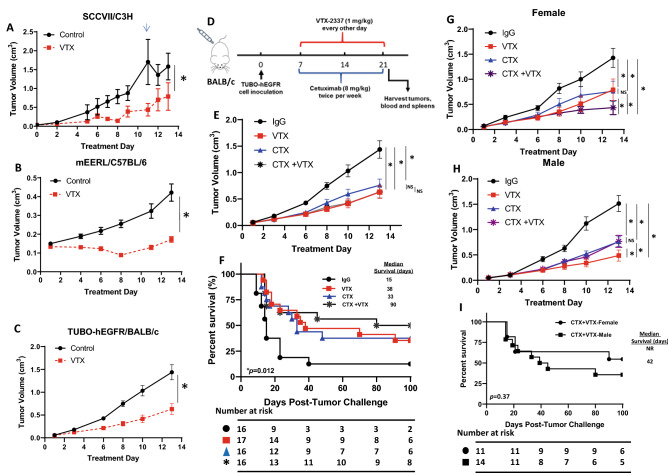
VTX-2337 shows an anti-tumor effect in vivo. (**A**–**C**) C3H mice bearing SCCVII tumors (**A**), C57BL/6 mice bearing mEERL (**B**), and BALB/c mice bearing TUBO-hEGFR tumors (**C**) were treated with PBS (Control) or VTX-2337 (VTX) and tumor growth was measured. Arrow in (**A**) represents the time at which tumor growth in a control-treated mouse reached euthanasia criteria. N = 5–9 mice/treatment group. (**D**) VTX and cetuximab (CTX) treatment schema. (**E**–**I**) BALB/c mice (n = 10 mice/group, 5 male/5 female) bearing TUBO-hEGFR tumors were treated as in (**D**) with IgG + PBS used as controls. Separate tumor growth graphs are shown for male (**F**) and female (**G**) mice from E. Error bars represent standard error of the mean. (**H**–**I**) Shown are Kaplan Meier curves comparing overall and median survival of all mice (**H**) and CTX + VTX-treated mice from H separated into female and male (I). *p < 0.05. NS: non-significant (Linear regression).

Given the effect of VTX on the secretion of cytokines involved in anti-tumor immune responses (Fig. 3), we investigated the anti-tumor effect of VTX in 3 syngeneic tumor mouse models. Treatment of VTX (1 mg/kg i.p. every other day) in female C3H mice bearing poorly immunogenic SCCVII tumors (Fig. 4A), female C57Bl/6 mice bearing HPV + mEERL tumors (Fig. 4B) and in female BALB/c mice bearing TUBO-hEGFR tumors (Fig. 4C) significantly suppressed tumor growth over the course of 2 weeks of treatment compared to control treatment. We next evaluated the anti-tumor effect of VTX in combination with CTX. In this case we utilized the TUBO-hEGFR/BALB/c mouse model. TUBO-hEGFR cells were genetically engineered to express human EGFR as a tool to study the immunotherapeutic effects of CTX since CTX can only bind to human EGFR and not murine EGFR^23^. One week after tumor inoculation into both male and female mice (n = 10, 5 female + 5 male) and after tumors were formed, control treatments (IgG + PBS) and VTX with and without CTX (2 mg/kg i.p. twice per week) were administered as illustrated in Fig. 4D. VTX + CTX significantly suppressed tumor growth compared to control (Fig. 4E). There was no enhanced anti-tumor effect of VTX + CTX observed compared to each agent alone (Fig. 4E). After the end of treatment (2 weeks) significant survival differences were observed among the treatment groups (Fig. 4F). VTX + CTX-treated mice survived significantly longer (median survival = 90 days) than mice treated with VTX (median survival = 38 days), CTX (median survival = 33 days) or control (median survival = 15 days) (Fig. 4F) suggesting a synergistic effect on survival outcomes. When mice were separated by sex, a partial but significant protective effect of VTX on CTX-induced tumor growth inhibition was observed in males compared to females (Fig. 4G,H). Survival results showed an advantage of VTX + CTX on survival in female mice (median survival = not reached) but not in male mice (median survival = 42 days) (Fig. 4I) or female ovariectomized (OVX) mice (Supplementary Fig. 1) although the difference in overall survival by log-rank test was not significant (Supplementary Fig. 1). These results suggest that sex/estrogen levels may affect the anti-tumor efficacy VTX + CTX.

### VTX-2337 in combination with CTX triggers immune cell recruitment

**Figure 5:**
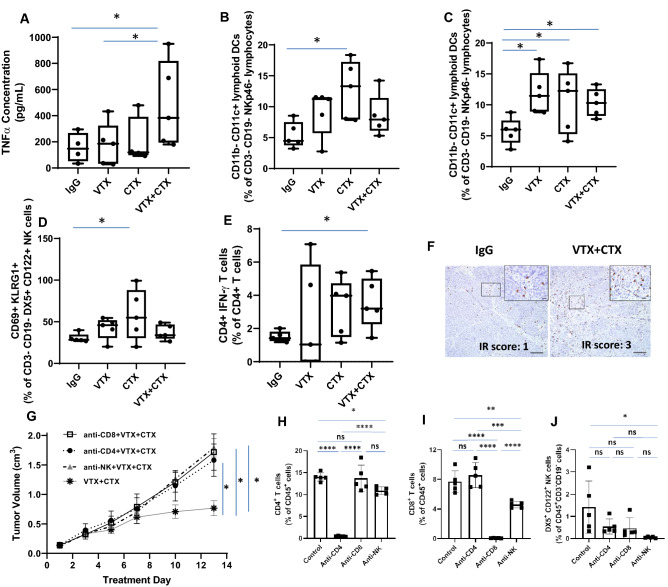
VTX-2337 and Cetuximab increase immune cell infiltration. Blood, tumors and spleens were harvested from a subset of mice (n = 4–5) one day after drug treatment and analyzed for serum concentrations of TNFα (**A**), and tumor (**B**) and splenic (**C**) lymphoid DCs, CD69 + NK cells (**D**) and IFNγ + CD4 + T cells (**E**) using flow cytometry. (**F**) Images represent CD8 immunostaining in control (left image) and VTX + CTX (right image)-treated tumors. Bars = 100 µm (inset bars = 20 µm). (**G**–**J**) Female BALB/c mice (n = 9–10 mice/group) bearing TUBO-hEGFR tumors were treated with VTX in combination with CTX with or without anti-CD4, anti-CD8 or anti-asialo-GM1 (anti-NK) and tumor growth (**G**) was measured. Spleens were analyzed by flow cytometry for validation of CD4^+^ T cell (H), CD8^+^ T cell (**I**) and NK cell (**J**) depletion. *p < 0.05, **p < 0.01, ***p < 0.001, ****p < 0.0001, ns: not significant (One-way ANOVA, Tukey’s multiple comparison test). Error bars represent standard deviation from the mean.

Levels of 23 cytokines/chemokines from the sera and levels of select immune cells from tumors and spleens from a subset (n = 4–5) of mice from each treatment group were measured immediately after the 2 week drug treatment period. Of the 23 cytokines/chemokines, only TNFα was increased in VTX+ CTX-treated mice compared to the other treatment groups (Fig. 5A). Tumor (Fig. 5B) and splenic (Fig. 5C) lymphoid DCs, CD69+ NK cells (Fig. 5D) and IFNγ+ CD4+ T cells (Fig. 5E) were significantly increased in CTX-treated tumors compared to controls but there was no further increase in VTX+ CTX-treated tumors compared to CTX alone (Fig. 5B–E). VTX and VTX+ CTX significantly increased splenic lymphoid DCs (Fig. 5C). No differences were observed among the treatment groups for total NK cells, myeloid DCs or neutrophils (data not shown). Although VTX+ CTX-treated tumors did not show a significant increase in percentages of IFNγ+ CD8+ T cells compared to control (data not shown), all VTX-treated tumors appeared to have a relative increase in CD8 immunostaining compared to controls (Fig. 5F). Depletion of CD4+ and CD8+ T cells completely reversed the anti-tumor effect of VTX+ CTX (Fig. 5G). Validation of T cell depletion from spleens is shown in Fig. 5H–I. NK cell depletion also completely reversed the anti-tumor effect of VTX+ CTX (Fig. 5G). Validation of NK cell depletion showed that NK cells were appropriately depleted but percentages of CD4+ and CD8+ T cells were also significantly depleted (Fig. 5J). Altogether, these results suggest that NK, CD4+ and CD8+ cells may all contribute to the anti-tumor effect of VTX + CTX.

## Discussion

Altogether, our studies implicate the role of T cell activation in the mechanism of action of VTX + CTX. Additionally, we report for what we believe is the first time, the significant association between TLR8 protein expression with CD8 expression and favorable survival outcomes in HNSCC patients (Fig. 2). This confirms the possible prognostic value of TLR8 in HNSCC patients and should be further pursued. Unexpectedly, we found that NK cell depletion was equally as effective as CD4+ and CD8+ T cell depletion at reversing the anti-tumor effect of VTX + CTX (Fig. 5G). VTX has been reported to be a NK cell activator^12,14,15^, although our in vitro co-culture assays do not support VTX alone as a NK cell activator (Fig. 3I). VTX however did enhance CTX-induced NK cell activation (Fig. 3I) which is a major reason why this drug combination was of interest for HNSCC therapy. It is possible that VTX + CTX-mediated NK cell activation may promote DC-NK cell crosstalk which would further enhance anti-tumor T cell responses compared to either drug alone. This is supported by the observation that NK cell depletion resulted in a significant decline in percentages of both CD4+ (Fig. 5H) and CD8+ T cells (Fig. 5I) in VTX + CTX-treated mice. TLR8 agonists are also known to trigger inflammasome activity in DCs, resulting in IL-1β secretion which can trigger NK cell activity^12^. The ability of VTX to trigger IL-1β secretion from PBMCs (Fig. 3C) supports the contribution of inflammasome activity in VTX-mediated immune response.

Despite the clear evidence of VTX + CTX-mediated anti-tumor immune response observed from the present work and from others, the obvious question is why did VTX fail to enhance CTX-based therapy in the Active 8 clinical trial^17^? Post-hoc analysis of this trial revealed that the only patients that benefitted from the therapy were those that experienced injection-site reactions (p = 0.023) and those with HPV + disease (p = 0.046)^17^. With regard to HPV status, it is not clear how HPV infection affects TLR8 expression and activity. Previous studies have reported that HPV 16 infections were significantly associated with an increase in expression of endosomal TLRs (TLR3, TLR7, TLR8 and TLR9) in human endocervical specimens^24^; and HPV 6b/11 E7 loading in mouse pDCs could promote the transcriptional level of TLR7 and TLR9^25^. This may explain why patients with HPV + disease benefitted from VTX + CTX-based therapy (17). On the other hand, studies suggest that HPV infection may suppress TLR signaling since the HPV16 E6 protein was found to inhibit TLR9 signaling although this finding was not observed with HPV18 E6^26^. It is likely that the effect of HPV infection on TLR expression/activity may depend on the HPV strain, cell type and TLR in question.

Our studies have raised the question of how EGFR status may be involved in tumor response to TLR8 agonists. Based on our results (Fig. 2C,D), would TLR8 agonists be more beneficial in low/no EGFR-expressing HNSCC patients? Would patients with high EGFR expression be more likely to benefit from CTX in an attempt to mimic a low/no EGFR phenotype in combination with TLR8 agonists? These questions cannot be readily answered since it is unclear if EGFR status was considered for the Active 8 clinical trial. More importantly, we found interesting gender differences in tumor response to VTX + CTX which may have profound implications on the ongoing use of this drug. Female mice were highly responsive to VTX + CTX while male mice were not (Fig. 4G–I). Additionally, when female mice were ovariectomized, the anti-tumor effect of VTX + CTX appeared to be abolished (Supplementary Fig. 1). It is not clear in this case why this sex or hormone difference exists, but studies have shown that the expression of TLR8 is positively regulated by estrogen due to the presence of an estrogen response element (ERE) proximal to the TLR8 genetic locus^27,28^. In fact, estrogen increases the expression of all the endosomal TLRs (TLR3,7,8,9)^27^ and endosomal TLR-mediated response of pDCs was found to be regulated by estrogen^29^. Perhaps the increased TLR8 expression/activity in females results in a greater ability for VTX to increase DC activity, enhance CTX-induced NK activity and trigger robust anti-tumor immune responses. This may explain the perceived failure of the Active 8 trial since the majority of subjects were male (85%)^17^ with the remainder probably being post-menopausal females although the age range of female patients was not reported. In a previous clinical study, treatment of post-menopausal women (ages 46–59 years) with 17β-estradiol dramatically enhanced TLR7 and TLR9 agonist-induced stimulation of pDCs^29^. It would be interesting to further study if postmenopausal female HNSCC patients would benefit from TLR8 agonist-based therapy with estrogen supplementation. Nevertheless, more attention should be placed in this area of sex hormone effects, endosomal TLR activity and survival outcomes in cancer patients, given prior intriguing findings on sex-specific effects of TLR8 in inflammatory disorders^27–32^, to determine how best to utilize motolimod and other endosomal TLR agonists for cancer therapy.

Lastly, it is important to mention that there is much controversy surrounding the functionality of TLR8 in mice and the use of mouse models to study TLR8 agonists. It was believed that TLR8 is non-functional in mice^33,34^ however this belief has been challenged by a number of recent studies that demonstrate the functionality of murine TLR8^35–38^. Additionally, our results clearly demonstrate that TLR8 is functional in mice since the anti-tumor activity of VTX2337 can be observed in 3 different mouse models (Fig. 4A–C).

## Conclusions

In conclusion, our results give further insight into the anti-tumor immune response associated with VTX + CTX for HNSCC therapy and highlights the need for further investigation of this drug combination with emphasis on how sex differences may affect this drug combination and other cancer immunotherapeutic agents.

## Supplementary Information

Supplementary Information.

## Data Availability

Datasets from the TCGA (TCGA_HNSC_exp_HiSeqV2-2015-02-24) are publicly available. The datasets generated during and/or analyzed during the current study are available from the corresponding author on reasonable request.

## Abbreviations

TLR: Toll-like receptor
VTX: VTX-2337/motolimod
NK: Natural killer
DC: Dendritic cell
pDC: Plasmacytoid dendritic cell
MYD88: Myeloid differentiation factor 88
TMA: Tissue microarray
HPV: Human papilloma virus
CTX: Cetuximab
OVX: Ovariectomized
ADCC: Antibody-dependent, cell-mediated cytotoxicity
EGFR: Epidermal growth factor receptor
HNSCC: Head and neck squamous cell carcinoma
IL-1β: Interleukin-1 beta
PR: Partial response
PD: Progressive disease
SD: Stable disease
PFS: Progression-free survival
R/M: Recurrent and metastatic
TCGA: The Cancer Genome Atlas
EXTREME: ERBITUX in first-line Treatment of REcurrent or MEtastatic head and neck cancer
