# Supplementary material for: The anti-tumor effects of cetuximab in combination with VTX-2337 are T cell dependent
Source: Sci Rep. 2021 Jan 15;11:1535. doi: 10.1038/s41598-020-80957-z (PMC7810827; doi:10.1038/s41598-020-80957-z)
Supplement: Supplementary file 1 — Supplementary Information. [file 41598_2020_80957_MOESM1_ESM.pdf]

Supplementary Information

The anti-tumor effects of cetuximab in combination with VTX-2337 are T cell dependent

Yinwen Cheng[1,2,3], Nicholas Borcherling[2,3,4], Ayomide Ogunsakin[5], Caitlin D. Lemke-Miltner[3,6], Katherine N. Gibson-Corley[2,3], Anand Rajan[2], Allen Choi[2], Wattawan Wongpattaraworakul[2,3,7], Carlos H. Chan[3,8], Aliasger K. Salem[1,3,8], George J. Weiner[3,6], Andrean L. Simons[1,2,3,7*]

[1]Interdisciplinary Graduate Program in Human Toxicology, University of Iowa, Iowa City, IA, [2]Department of Pathology, University of Iowa, Iowa City, IA, [3]Holden Comprehensive Cancer Center, University of Iowa, Iowa City, IA, [4]Iowa Medical Scientist Training Program, Carver College of Medicine, University of Iowa, Iowa City, IA, [5]Department of Biochemistry, Lincoln University, Lincoln University, PA, [6]Department of Internal Medicine, University of Iowa, Iowa City, IA, [7]Department of Oral Pathology, Radiology and Medicine, College of Dentistry, University of Iowa, [8]Department of Surgery, [8]Division of Pharmaceutics and Translational Therapeutics, College of Pharmacy, University of Iowa, Iowa City, IA.

***Corresponding author:**
Andrean L. Simons, PhD
Associate Professor
Department of Pathology
1161 Medical Laboratories
University of Iowa
Iowa City, IA 52242
Phone: (319) 384-4450
Fax: (319) 335-8453
Email: andrean-simons@uiowa.edu

Supplemental Methods

Ovariectomized (OVX) and female SHAM mice (n=5 mice/group) were administered VTX+CTX and control (IgG+PBS) as already described for Figure 4D. Tumor growth was measured 3 times per week using Vernier calipers and mouse weights were recorded. Tumor volume was calculated using the following formula: volume = $width^2$ x length, where length was the longest dimension of the tumor and width was the dimension perpendicular to length. When the diameter of tumors reached 15 mm in any direction, mice were euthanized via CO_2 asphyxiation. Tumor growth curve and mice survival curves were plotted.

Supplementary Table 1: Correlation between TLR8 expression and clinical outcomes in HNSCC patients

Patient characteristics	Total (n=522)	TLR8 Expression			*p*-value
		Low *TLR8* (n=115)	Medium *TLR8* (n=316)	High *TLR8* (n=91)	
**Gender**					
Female	136	18.3% (N=21)	25% (N=79)	39.6% (N=36)	*p=0.002*
Male	386	81.7% (N=94)	75% (N=237)	60.4% (N=55)	
**Median age at diagnosis (Range)**		58 (24-85)	61 (19-88)	64 (26-90)	
Female	136	60 (32-85)	63 (24-87)	66 (36-99)	*p=0.97*
Male	386	59 (24-85)	59 (19-88)	61 (26-87)	
**Clinical stage**					
I	20	1.7% (2)	4.1% (13)	5.5% (5)	
II	98	22.6% (26)	17.4% (55)	18.7% (17)	*p=0.59*
III	105	20.9% (24)	20.9% (66)	16.5% (15)	
IV	285	50.4% (58)	55.7% (176)	56.0% (51)	
Unknown	14	4.3% (5)	1.9% (6)	3.3% (3)	
**Primary therapy outcome success**					
Complete remission/response	374	70.4% (81)	69.9% (221)	79.1% (72)	
Partial remission/response	6	1.7% (2)	1.3% (4)	0% (0)	
Persistent disease	4	1.7% (2)	0.6% (2)	0% (0)	*p=0.81*
Progressive disease	39	6.1% (7)	8.9% (28)	4.3% (4)	
Stable disease	6	0.9% (1)	1.3% (4)	1.1% (1)	
Unknown	93	19.1% (22)	18.0% (57)	15.4% (14)	
**Follow up treatment success**					
Complete remission/response	228	39.1% (45)	45.6% (144)	42.8% (39)	
Partial remission/response	5	0.9% (1)	1.3% (4)	0% (0)	
Persistent disease	12	1.7% (2)	1.3% (4)	6.6% (6)	*p=0.16*
Progressive disease	75	16.5% (19)	13.9% (44)	13.2% (12)	
Stable disease	7	1.7% (2)	0.9% (3)	2.2% (2)	
Unknown	195	40% (46)	37.0% (117)	35.2% (32)	
**HPV status**					
Negative	73	13.9% (16)	11.1% (35)	24.2% (22)	
Positive	39	6.1% (7)	7.6% (24)	8.8% (8)	*p=0.026*
Unknown	410	80% (92)	81.3% (257)	67.0% (61)	
**Radiation therapy**					
Yes	292	54.8% (63)	57% (180)	53.8% (49)	
No	160	30.4% (35)	30% (95)	33% (30)	*p=0.97*
Unknown	70	14.8% (17)	13% (41)	13.2% (12)	
**Targeted molecular therapy**					
Yes	149	27% (31)	27.8% (88)	33% (30)	
No	260	47% (54)	50.9% (161)	49.4% (45)	*p=0.59*
Unknown	113	26% (30)	21.2% (67)	17.6% (16)	

Supplementary Figure 1

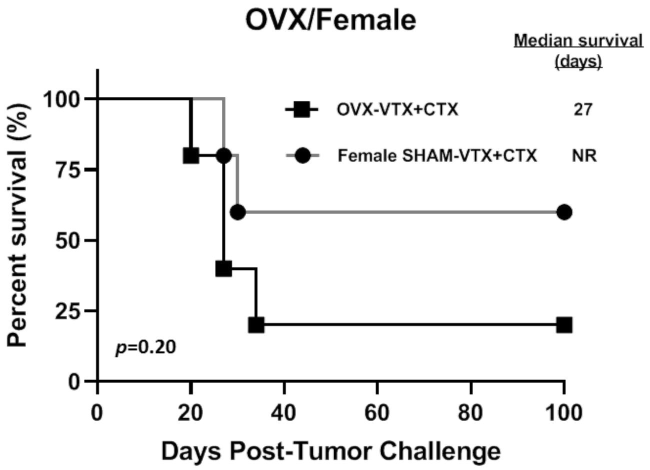

Number at risk

●	5	5	4	4	4	3
■	5	5	2	2	2	1

Supplementary Figure 1. Response to VTX-2337+Cetuximab is sex-specific. Shown are Kaplan Meier curves comparing overall and median survival of VTX+CTX-treated SHAM and ovariectomized (OVX) TUBO-hEGFR tumor-bearing female mice. Mice were treated as illustrated in Figure 4D.
